# Supplementary material for: Salmonella enterica Serovar Typhi Induces Host Metabolic Reprogramming to Increase Glucose Availability for Intracellular Replication
Source: Int J Mol Sci. 2021 Sep 16;22(18):10003. doi: 10.3390/ijms221810003 (PMC8467381; doi:10.3390/ijms221810003)
Supplement: Supplementary file 1 [file ijms-22-10003-s001.zip › ijms-1365911-SI.pdf]

Supplementary Material

Salmonella Typhi Induces Host Metabolic Reprogramming to Increase Glucose Availability for Intracellular Replication

Jingting Wang[1,2], Shuai Ma[1,2], Wanwu Li[1,2], Xinyue Wang[1,2], Di Huang[1,2], Lingyan Jiang[1,2,*] and Lu Feng [1,2,*]

[1] The Key Laboratory of Molecular Microbiology and Technology, Ministry of Education, Nankai University, Tianjin, China
[2] TEDA Institute of Biological Sciences and Biotechnology, Tianjin Key Laboratory of Microbial Functional Genomics, Nankai University, Tianjin, China
* Correspondence: Lu Feng: fenglu63@nankai.edu.cn; Lingyan Jiang: jianglingyan@nankai.edu.cn

Table S1. Bacterial strains and plasmids used in this study.

Strain or plasmid	Genotype or description	Source
Strains		
Wild-type	*Salmonella enterica* serovar Typhi Ty2	Lab collection
Δ*lldP*	Wild-type *lldP*::Cm; Cm^R	This study
Δ*ptsG manXYZ glk*	Wild-type *ptsG*::Km, *manXYZ*::Cm, *glk*::Km; Cm^R	This study
Δ*pgtP*	Wild-type *pgtP*::Cm; Cm^R	This study
Plasmids		
pSim17	For generating mutant strain with λ Red recombinase system; Bs^R	Lab collection
pKD3	For λ Red recombination; Cm^R	Lab collection
pKD4	For λ Red recombination; Km^R	Lab collection
pCP20	Temperature-sensitive replicon expressing the FLP gene to remove antibiotic resistance of mutant strains; Ap^R	Lab collection

Table S2. Oligonucleotides used in this study

Target gene		Primer sequence (5′-3′)
		**Primer used for construction of the mutants**
ptsG	F	CCGAATGGCTGCCTTAATACTCCCCAACATCATTACTGC GTGTAGGCTGGAGCTGCTTC
	R	AACGTAGAAAAGCACAAATACTCAGGAGCACTCTCAATT ATGGGAATTAGCCATGGTCC
lldP	F	GAGAGGCAATCTCGTCTGACAGGCGTTTTGGCATCACAA GTGTAGGCTGGAGCTGCTTC
	R	GATTCATCCCATTATGCGTGTGGTTCTCAGGAGACCTGCA ATGGGAATTAGCCATGGTCC
manXYZ	F	AAAAACGGGGCCGTTTGGCCCCGGTAGTGTACAACAGCC GTGTAGGCTGGAGCTGCTTC
	R	GTCAAGTTGATGTGTTGACAATAATAAAGGAGGTAGCAA ATGGGAATTAGCCATGGTCC
glk	F	GACAAAGACTTATTTTGACTTTAGCGGAGTAGTAGAAGA GTGTAGGCTGGAGCTGCTTC
	R	TTTTGTAGGCCGGATAAGGCGTTTATGCCGCCGTCTGAC ATGGGAATTAGCCATGGTCC
pgtP	F	ACAATGTCGGCGCTTCTGTTCCCCGGGAAGGCTAATCGT GTGTAGGCTGGAGCTGCTTC
	R	TTGCTGTTGTTGTCAGCCAGATTGAAAGTAACTGTAATA ATGGGAATTAGCCATGGTCC
		**Primer used for identification of the mutants**
ptsG	F	ACAGGGCAGCTATGCGCTGGTCGAT
	R	GCGCGTAAAGTTCACCGCCACAAAAG
lldP	F	TTCAGCGGTTGCACGATATTCTGTTC
	R	ATGCCATTGCCTTTTCACTTCCCT
manXYZ	F	CGCTCCGTGGCTGGTTACGTTGTTA
	R	ACAACTGGTTTTTGGCAAAGATTTA
glk	F	ATAATCAGCGCAGGGAGCGACAGCA
	R	CCTGTTTTCGCCTGAGTAATCACCA
pgtP	F	TACATACCTTTCATACTTCAAGTGG
	R	GAGGTGATGAAGTTTTCATCTTGTC

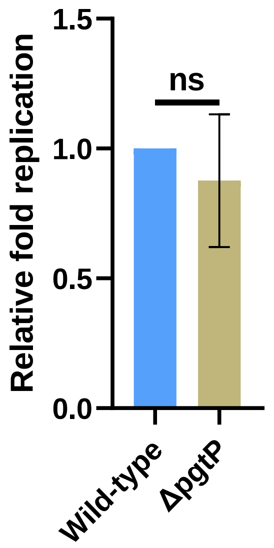

Figure S1. Replication of wild-type *Salmonella enterica* serovar Typhi (*S*. Typhi) and *ΔpgtP* in THP-1 cells. The data were obtained from three independent experiments and analyzed using Student's *t*-test. ns, not significant ($P > 0.05$).

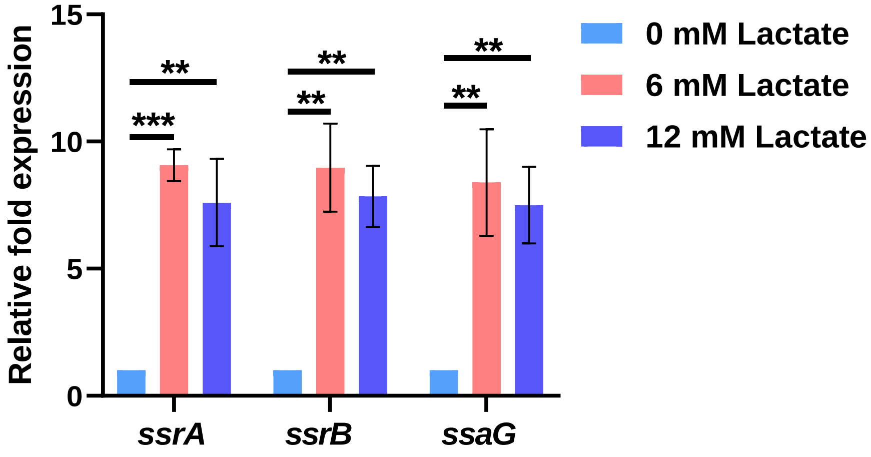

Figure S2. Quantitative reverse transcription polymerase chain reaction analysis of SPI-2 gene mRNA levels in 0, 6, or 12 mM sodium lactate-treated *Salmonella enterica* serovar Typhi in N-minimal medium. The data were obtained from three independent experiments and analyzed using one-way ANOVA. ** $P < 0.01$; *** $P < 0.001$.
